# Improved kidney function in patients who switch their protease inhibitor from atazanavir or lopinavir to darunavir

**DOI:** 10.1097/QAD.0000000000001353

**Published:** 2017-02-01

**Authors:** Sophie Jose, Mark Nelson, Andrew Phillips, David Chadwick, Roy Trevelion, Rachael Jones, Deborah I. Williams, Lisa Hamzah, Caroline A. Sabin, Frank A. Post

**Affiliations:** a Research Department of Infection and Population Health, University College London; b Chelsea and Westminster NHS Foundation Trust, London; c South Tees Hospitals NHS Foundation Trust, Middlesbrough; d HIV I-Base, London; e Brighton and Sussex University Hospitals, Brighton; f Kings College Hospital NHS Foundation Trust, London, UK.

**Keywords:** atazanavir, darunavir, HIV, kidney, lopinavir

## Abstract

**Objective:**

Atazanavir (ATV) and lopinavir (LPV) have been associated with kidney disease progression in HIV positive patients, with no data reported for darunavir (DRV). We examined kidney function in patients who switched their protease inhibitor from ATV or LPV to DRV.

**Design:**

Cohort study.

**Methods:**

Data were from the UK CHIC study. We compared pre and post switch estimated glomerular filtration rate (eGFR) slopes (expressed in ml/min per 1.73 m^2^ per year) in all switchers and those with rapid eGFR decline (>5 ml/min per 1.73 m^2^ per year) on ATV or LPV. Mixed-effects models were adjusted for age, gender, ethnicity, eGFR at switch and time updated CD4^+^ cell count, HIV RNA and cumulative tenofovir (tenofovir disoproxil fumarate) exposure.

**Results:**

Data from 1430 patients were included. At the time of switching to DRV, median age was 45 years, 79% were men, 76% had an undetectable viral load, and median eGFR was 93 ml/min per 1.73 m^2^. Adjusted mean (95% confidence interval) pre and post switch eGFR slopes were −0.84 (−1.31, −0.36) and 1.23 (0.80, 1.66) for ATV (*P* < 0.001), and −0.57 (−1.09, −0.05) and 0.62 (0.28, 0.96) for LPV (*P* < 0.001). Stable or improved renal function was observed in patients with rapid eGFR decline on ATV or LPV who switched to DRV [−15.27 (−19.35, −11.19) and 3.72 (1.78, 5.66), *P* < 0.001 for ATV, −11.93 (−14.60, −9.26) and 0.87 (−0.54, 2.27), *P* < 0.001 for LPV]. Similar results were obtained if participants who discontinued tenofovir disoproxil fumarate at the time of switch were excluded.

**Conclusions:**

We report improved kidney function in patients who switched from ATV or LPV to DRV, suggesting that DRV may have a more favourable renal safety profile.

## Abstract 2

Supplemental Digital Content is available in the text

## Introduction

Antiretroviral therapy (ART) allows people living with HIV to achieve a normal life expectancy [1]. Widespread use of ART has resulted in ageing HIV populations in whom comorbidities such as chronic kidney disease (CKD), hypertension, diabetes, and cardiovascular disease are increasingly prevalent, diagnosed earlier than in the general population, and important causes of death [2–4]. The potential contribution of ART to the burden of CKD has been the focus of several large observational cohort studies; tenofovir disoproxil fumarate (TDF), atazanavir (ATV) and lopinavir (LPV) have been associated with incident CKD and CKD progression in these studies [5–7], although the magnitude of the reported associations was relatively modest (14–16%, 20–21%, and 8–11% increased incidence of CKD with each additional year of exposure to TDF, ATV, and LPV) [5,6]. A possible explanation for these observed associations may be that TDF can give rise to acute tubular injury and ATV to kidney stone formation and interstitial nephritis [8–10]. In addition, infrequent reports have linked LPV and ritonavir (RTV) to acute kidney injury and stone formation [11–13]. By contrast, long-term renal safety data for darunavir (DRV) are sparse and it remains unclear whether the observed association of ATV and LPV with CKD reflects a protease inhibitor class effect, or whether DRV is a well tolerated treatment option for patients with, or at risk of CKD and kidney disease progression. RTV is an inhibitor of P-glycoprotein, the cytochrome P450 system that boosts the plasma concentrations of LPV, ATV, and DRV, and several renal tubular transporters involved in the clearance of drugs and secretion of creatinine [14]. Reduced tubular secretion of creatinine results in small increases in plasma creatinine and, thereby, modest reductions in estimated glomerular filtration rate (eGFR) [14]. Such changes, which are typically acute (<4 weeks) in onset, nonprogressive during follow-up and nontreatment limiting [15], may affect the ability to accurately study kidney disease progression in patients who receive protease inhibitor therapy. This confounding effect, however, can be minimized by studying serial eGFR in patients who switch between boosted protease inhibitor.

We analysed eGFR slopes in patients who switched their RTV-boosted protease inhibitor from ATV or LPV to DRV and, based on our earlier work [16], hypothesised that there would be no difference between pre and post switch eGFR slope. We performed several planned subgroup analyses to examine the effect of TDF coexposure and the presence of rapid eGFR decline or CKD prior to switching to DRV.

## Methods

We used data from the UK CHIC Study of HIV-positive people who have accessed HIV outpatient care at any of the participating clinics since 1996 [17]. Individuals were included if they switched from either ATV or LPV to DRV (each boosted with RTV) at any point during follow-up and had at least 6-months exposure to and more than two serum creatinine measurements on the relevant protease inhibitor pre and post switch. We allowed DRV to be started no more than 7 days after discontinuation of ATV or LPV. Study participants were followed from the time of starting either ATV or LPV to the earliest of DRV discontinuation, the date of last follow-up in UK CHIC, or 31 December 2013.

Clinical characteristics at the time of protease inhibitor switch were described for those that switched from ATV or LPV to DRV. The CKD-EPI formulation was used to convert creatinine measurements to eGFR [18]. To estimate and compare the rate of eGFR change per year before and after protease inhibitor switch, piecewise mixed effects models with random intercept and time terms were fitted with eGFR as the dependent variable. The effect of time was allowed to change pre and post protease inhibitor switch, thereby allowing estimation of pre and post switch eGFR slopes. Mixed effects models were adjusted for the following factors: age at switch, gender, ethnicity (white, black, other/unknown), eGFR at switch and time updated CD4^+^ cell count, HIV RNA [undetectable (<50 copies/ml), detectable] and cumulative TDF use (where appropriate). Separate models were fitted for those on ATV or LPV. eGFR slopes were also estimated in a group of individuals exposed to either ATV or LPV who did not switch to DRV, to provide context to our findings. These individuals were also required to have at least 6- months exposure to the protease inhibitor in question with at least two serum creatinine measurements during this time.

Rapid eGFR decline was defined as a decline in eGFR of more than 3 and more than 5 ml/min per 1.73 m^2^ per year as estimated from unadjusted pre switch eGFR slopes in the mixed effects model. Subgroup analyses were performed that restricted analyses to those with rapid eGFR decline, restricted to those with an eGFR less than 60 ml/min per 1.73 m^2^ prior to switching, excluded those who also discontinued TDF at the time of protease inhibitor switch and estimated eGFR slopes separately for those with and without TDF use prior to switching. In sensitivity analyses, we estimated pre and post switch eGFR slopes after excluding eGFR measurements in the first 4 weeks of starting the ATV, LPV, and DRV-containing regimens, excluding the last eGFR prior to switch, and restricting eGFR measurements to only 2 year of follow-up either side of the protease inhibitor switch. To provide context, eGFR slopes were also estimated for individuals in the UK CHIC Study who commenced ATV or LPV for at least 6 months and who did not switch to DRV, provided they had at least two creatinine measurements during the period of ATV or LPV exposure. For any individual satisfying these criteria at more than one time point, the first episode was used.

## Results

A total of 2667 people switched from either LPV/RTV or ATV/RTV to DRV/RTV during UK CHIC follow-up. Of these, 1894 had at least 6-months follow-up on the protease inhibitor pre and post switch and 1430 also had at least two creatinine measurements available during the pre and post switch periods. The characteristics of the 577 patients who switched from ATV to DRV and the 853 patients who switched from LPV to DRV were broadly similar (Table 1). The earliest switch in the UK CHIC data set occurred in 2005, and the median year of protease inhibitor switch was 2010. The median age at switch was 45 years; ART exposure was extensive (median duration 10.1 years), 920 study participants (64%) were on TDF, and 76% had an undetectable viral load at the time of switching. A total of 647 (45.2%) individuals made changes to another aspect of their ART regimen at the time of the protease inhibitor switch. A nucleoside reverse transcriptase inhibitor (NRTI) was switched, discontinued, or added to the regimens of 158 (11.0%), 352 (24.6%), and 51 (3.6%) people, respectively; 236 (16.5%) discontinued TDF. Changes involving a non-nucleoside reverse transcriptase inhibitor (NNRTI) were made in 183 (12.8%) individuals and 133 (9.3%) changed another ARV, such as an entry or fusion inhibitor. The median (interquartile range, IQR) eGFR at switch was 90 (74, 106) ml/min per 1.73 m^2^ in the ATV group and 94 (79, 107) ml/min per 1.73 m^2^ in the LPV group. The median (IQR) duration of ATV and LPV exposure was 3.1 (1.6, 4.7) and 4.1 (2.2, 6.8) years, and the median duration of DRV exposure 2.6 (1.6, 3.8) and 3.0 (2.0, 4.2) years, respectively.

**Table 1:** Patient characteristics at switch from atazanavir (/r) or lopinavir (/r) to darunavir (/r).

	Lopinavir switch	Atazanavir switch	Any switch
Characteristics	*N* = 853	*N* = 577	*N* = 1430
Age			
Years	45 (40, 51)	45 (39, 51)	45 (40, 51)
Sex			
Male	671 (78.7)	463 (80.2)	1134 (79.3)
Female	182 (21.3)	114 (19.8)	296 (20.7)
Ethnicity			
White	593 (69.5)	407 (70.5)	1000 (69.9)
Black	204 (23.9)	123 (21.3)	327 (22.9)
Other/unknown	56 (6.6)	47 (8.2)	103 (7.2)
Exposure			
Sex between men	538 (63.1)	375 (65.0)	913 (63.9)
Heterosexual	261 (30.6)	166 (28.8)	427 (29.9)
Other/unknown	54 (6.3)	36 (6.2)	90 (6.3)
Year			
2005–2009	399 (46.8)	248 (43.0)	767 (45.2)
2010–2013	454 (53.2)	329 (57.0)	924 (54.8)
CD4^+^ cell count			
cells/μl	517 (349, 728)	510 (367, 705)	513 (359, 719)
Viral load			
Detectable (>50)	189 (22.2)	151 (26.2)	340 (23.8)
Undetectable (<50)	661 (77.8)	425 (73.8)	1086 (76.2)
NRTI backbone			
TDF-containing	501 (58.7)	419 (72.6)	920 (64.3)
non-TDF	204 (23.9)	115 (19.9)	319 (22.3)
None	148 (17.4)	43 (7.5)	191 (13.4)
Other PI use			
No	722 (84.6)	619 (91.7)	1251 (87.5)
Yes	131 (15.4)	48 (8.3)	179 (12.5)
NNRTI use			
No	750 (87.9)	534 (92.6)	1284 (89.8)
Yes	103 (12.1)	43 (7.5)	146 (10.2)
eGFR			
<60	57 (6.7)	72 (12.5)	129 (9.0)
60–75	106 (12.4)	90 (15.6)	196 (13.7)
75–90	202 (23.7)	127 (22.0)	329 (23.0)
>90	488 (57.2)	128 (49.9)	776 (54.3)
ml/min per 1.73 m^2^	94 (79, 107)	90 (74, 106)	93 (77, 107)

eGFR, estimated glomerular filtration rate; NNRTI, nonnucleoside reverse transcriptase inhibitor; NRTI, nucleoside reverse transcriptase inhibitor; PI, protease inhibitor; TDF, tenofovir disoproxil fumarate. Data are shown as *N* (%) except age and CD4^+^ cell count which are shown as median (interquartile range).

The mean eGFR slope estimates of people who switched from ATV or LPV to DRV are shown in Table 2. A small annual decline in eGFR prior to switch was observed during exposure to ATV or LPV. The eGFR slopes improved significantly after these patients switched their protease inhibitor to DRV. Estimated eGFR slopes pre and post switch remained similar after adjusting for a-priori factors mentioned previously. A decline in eGFR was still observed prior to switch [−0.84 (−1.31, −0.36) and −0.57 (−1.09, −0.05) ml/min per 1.73 m^2^ per year for ATV and LPV], with improvement in eGFR post switch [1.23 (0.80, 1.66) and 0.62 (0.28, 0.96) ml/min per 1.73 m^2^ per year for ATV and LPV, respectively]. Declines in eGFR were also observed in 6955 study participants exposed to ATV or LPV for at least 6 months (with at least two eGFR measurements) who did not switch to DRV; −0.51 (-0.67, -0.34; ATV) and −0.76 (−0.94, −0.59; LPV) ml/min per 1.73 m^2^ per year, respectively.

**Table 2:** eGFR slope estimates in individuals who switch from either atazanavir (/r) or lopinavir (/r) to darunavir (/r).

		Mean change in eGFR per year (95% CI)		
Regimen	*N*	Pre switch	Post switch	*P* value^*^
A. All switchers				
Unadjusted estimates				
All switchers	1430	−0.87 (−1.21, −0.53)	0.51 (0.25, 0.77)	<0.001
Atazanavir	577	−0.94 (−1.38, −0.49)	0.90 (0.47, 1.34)	<0.001
Lopinavir	853	−0.78 (−1.29, −0.28)	0.27 (−0.05, 0.59)	<0.001
Adjusted estimates^**^				
All switchers	1430	−0.67 (−1.03, −0.31)	0.86 (0.59, 1.12)	<0.001
Atazanavir	577	−0.84 (−1.31, −0.36)	1.23 (0.80, 1.66)	<0.001
Lopinavir	853	−0.57 (−1.09, −0.05)	0.62 (0.28, 0.96)	<0.001

		Mean change in eGFR per year (95% CI)		
Regimen	*N*	Pre switch	Post switch	*P* value^*^
B. Excluding individuals who discontinued TDF at the time of PI switch^**^				
Atazanavir	443	−0.45 (−1.00, 0.09)	0.79 (0.30, 1.27)	0.002
Lopinavir	751	−0.44 (−1.03, 0.16)	0.62 (0.26, 0.99)	0.004

		Mean change in eGFR per year (95% CI)		
Regimen	*N*	Pre switch	Post switch	*P* value^*^
C. Patients who received TDF prior to switch (regardless of TDF use post switch)^†^				
Atazanavir	419	−1.27 (−1.80, −0.74)	1.37 (0.85, 1.88)	<0.001
Lopinavir	501	−1.26 (−1.58, −0.95)	0.33 (−0.07, 0.73)	<0.001

		Mean change in eGFR per year (95% CI)		
Regimen	*N*	Pre switch	Post switch	*P* value^*^
D. Patients who did not receive TDF prior to switch (regardless of TDF use post switch)^†^				
Atazanavir	158	−0.22 (−1.17, 0.72)	0.51 (−0.20, 1.22)	0.240
Lopinavir	351	0.20 (−1.25, 1.66)	0.52 (−0.04, 1.09)	0.695

Slopes are measured in ml/min per 1.73 m^2^ per year. CI, confidence interval; eGFR, estimated glomerular filtration rate; PI, protease inhibitor; TDF, tenofovir disoproxil fumarate.

^*^Pre vs. post switch eGFR slopes.

^**^Model includes age, sex, ethnicity, eGFR at switch, CD4^+^ count, undetectable viral load (yes/no) and cumulative TDF exposure.

^†^Model includes age, sex, ethnicity, eGFR at switch, CD4^+^ count and undetectable viral load (yes/no).

Exclusion of the 236 study participants on ATV or LPV who also discontinued TDF at the time of protease inhibitor switch resulted in more moderate but still significantly different pre and post switch eGFR slopes (Table 2b). When split by TDF use prior to switch, eGFR slope estimates for those who received ATV or LPV with TDF were similar to those in the main analysis, with a significant difference in pre and post switch estimates (Table 2c). By contrast, the pre and post switch slope estimates were not significantly different amongst study participants who received ATV or LPV without TDF (Table 2d). Sensitivity analyses excluding eGFR measurements in the first 4 weeks of both the pre and post switch regimens, excluding the last eGFR prior to switch and considering only 2-year follow-up either side of switching did not affect our results (data not shown).

We then analysed the changes in eGFR slopes in patients who switched to DRV with adverse renal profiles on ATV or LPV. A total of 205 (14.3%) study participants had eGFR decline more than 3 ml/min per 1.73 m^2^ per year, 92 (6.4%) had eGFR decline more than 5 ml/min per 1.73 m^2^ per year, and 129 (9.0%) patients had eGFR less than 60 ml/min per 1.73 m^2^ prior to switch. As expected, much steeper pre switch eGFR decline was observed in these patients as compared with the overall switch cohort (Table 3). Interestingly, the mean eGFR slopes dramatically improved post switch, with eGFR recovery during DRV exposure in several subgroups and stabilisation of renal function in the remainder. Similar results were obtained if the analyses were restricted to study participants with adverse eGFR profiles who did not discontinue TDF at the time of protease inhibitor switch (Supplementary Table, http://links.lww.com/QAD/B23).

**Table 3:** eGFR slope estimates in patients with rapid eGFR decline or eGFR more than 60 on atazanavir (/r) or lopinavir (/r).

		Mean change in eGFR per year (95% CI)		
Regimen	*N*	Pre switch	Post switch	*P* value^*^
Rapid eGFR decline (>3 ml/min per 1.73 m^2^): unadjusted estimates				
Atazanavir	94	−10.12 (−11.95, −8.29)	1.68 (0.16, 3.20)	<0.001
Lopinavir	111	−7.99 (−9.01, −6.96)	0.42 (−0.46, 1.31)	<0.001
Rapid eGFR decline (>3 ml/min per 1.73 m^2^): adjusted estimates^†^				
Atazanavir	94	−9.69 (−11.51, −7.88)	2.37 (1.08, 3.67)	<0.001
Lopinavir	111	−7.28 (−8.39, −6.18)	0.92 (0.03, 1.82)	<0.001
Rapid eGFR decline (>5 ml/min per 1.73 m^2^): unadjusted estimates				
Atazanavir	48	−15.25 (−19.22, −11.27)	2.91 (0.69, 5.12)	<0.001
Lopinavir	44	−13.17 (−15.72, −10.61)	0.51 (−0.99, 2.01)	<0.001
Rapid eGFR decline (>5 ml/min per 1.73 m^2^): adjusted estimates^†^				
Atazanavir	48	−15.27 (−19.35, −11.19)	3.72 (1.78, 5.66)	<0.001
Lopinavir	44	−11.93 (−14.60, −9.26)	0.87 (−0.54, 2.27)	<0.001
eGFR <60 ml/min per 1.73 m^2^: unadjusted estimates				
Atazanavir	72	−7.10 (−9.67, −4.53)	3.21 (1.65, 4.77)	<0.001
Lopinavir	57	−3.51 (−5.13, −1.90)	2.52 (0.44, 4.60)	<0.001
eGFR <60 ml/min per 1.73 m^2^: adjusted estimates^†^				
Atazanavir	72	−6.29 (−8.68, −3.90)	3.40 (1.82, 4.98)	<0.001
Lopinavir	57	−2.60 (−4.05, −1.15)	2.56 (0.40, 4.72)	<0.001

Slopes are measured in ml/min per 1.73 m^2^ per year. CI, confidence interval; eGFR, estimated glomerular filtration rate; PI, protease inhibitor; TDF, tenofovir disoproxil fumarate.

^*^Pre vs. post switch eGFR slopes.

^†^Model includes age, sex, ethnicity, eGFR at switch, CD4^+^ cell count, undetectable viral load (yes/no) and cumulative TDF exposure.

## Discussion

Contrary to our hypothesis, patients in the UK CHIC cohort who switched their protease inhibitor from ATV or LPV to DRV experienced small but statistically significant improvements in renal function. The improvements in eGFR slope were observed in patients who received ATV or LPV with TDF and much more pronounced in study participants who had experienced rapid eGFR decline or renal impairment prior to protease inhibitor switch. Our data suggest that ATV and LPV, especially when coadministered with TDF, may result in kidney injury and that switching these protease inhibitor to DRV may preserve renal function.

The effects of ATV and DRV on kidney function have been compared in several studies. Two randomized controlled clinical trials of ART-naïve patients who initiated ATV or DRV with RTV, TDF, and emtricitabine (FTC) reported similar changes in creatinine clearance or eGFR [19,20], and another trial a similar incidence (7/556 vs. 12/546) of grade 3 or 4 elevations in serum creatinine at 96 weeks [21]. A cross-sectional study reported similar proportions of patients on ATV and DRV having crystalluria (8.9 and 7.8%, respectively) [22]. By contrast, a Japanese retrospective study noted a considerably greater incidence of nephrolithiasis with ATV as compared with DRV [incidence rate 20.2 vs. 0.86 per 1000 person-years, hazard ratio 21.5 (95%confidence interval 2.9, 160)] [23]. The effects of LPV and DRV on the kidney have also been compared in a randomized controlled clinical trial. Patients in the ARTEMIS trial who received LPV or DRV with RTV and TDF/FTC experienced similar changes in creatinine clearance, renal adverse events and kidney stones [24]. Differences between clinical trial and cohort populations, with high-risk populations generally excluded from participating in clinical trials, and limited power to detect small differences in eGFR slope (<1 ml/min per 1.73 m^2^ per year) may potentially explain the disparate results between the randomized controlled trial data and our observational cohort data, underscoring the importance of both types of studies to detect potential adverse effects of ART on the kidney. The small sample size (*n* = 47) and the inclusion of study participants who received LPV (74%) or a non-TDF regimen (37%) may explain why our earlier study failed to show a change in eGFR slope following protease inhibitor switch to ATV [16]. Interestingly, improved kidney function (assessed by plasma cystatin C) has been reported in a small pilot study in which 13 study participants changed their ART regimen from LPV (or fosamprenavir) to DRV [25].

In our study, the majority of patients who received ATV or LPV without TDF had stable renal function pre switch and no significant improvement in eGFR post switch, suggesting that these drugs *per se* have limited nephrotoxic potential [14]. Several studies have noted an adverse effect of TDF and protease inhibitor coadministration on the kidney: in ACTG 5202, eGFR decline was reported in study participants who were randomized to ATV with TDF/FTC but not in those who received ATV with abacavir/lamivudine (ABC/3TC) or efavirenz with TDF/FTC or ABC/3TC [26], and in CCTG 578, study participants randomized to receive TDF with a protease inhibitor (rather than an NNRTI) experienced greater eGFR decline [27]. Furthermore, discontinuation of TDF may result in considerable improvement in renal function, with eGFR returning to pre-TDF values in approximately three quarters of patients [28].

We defined rapid eGFR decline using an average rate of decline over the period on ATV or LPV, based on the results of mixed-effects models and a minimum of 6-months exposure to these drugs. The definition of rapid progression proposed by D:A:D requires extensive (at least 3 year) follow-up and at least two creatinine measures per year, and was applied to individuals with eGFR at least 90 ml/min per 1.73 m^2^ in the original study [29]. When these more stringent criteria were applied to our study population, only 140 individuals met the definition of rapid eGFR decline. In their study, the authors acknowledge the potential for selection bias introduced by requiring a minimum of 3-year of follow-up and the exclusion of patients with eGFR 90 ml/min per 1.73 m^2^ or less at baseline. Indeed, the described changes in eGFR slope in our study are likely to be of greatest clinical significance in the group of participants who already had impaired renal function prior to initiating ATV or LPV.

Our study was well powered to examine pre and post switch differences in eGFR slope. By restricting the analyses to patients who received RTV-boosted protease inhibitor, we eliminated the effect of RTV on tubular creatinine secretion. However, some limitations need to be acknowledged. The UK CHIC study has incomplete data on the reasons for ART switches and no data on comorbidities and concomitant medications which could have affected eGFR. We had to exclude almost half of all study participants who switched from ATV or LPV to DRV because of insufficient eGFR data to construct slopes for comparison. As creatinine was measured in local laboratories, we are unable to exclude small intersubject differences in creatinine measurements. Finally, by nature of observational cohort studies, we are unable to exclude that bias or confounding may have affected our results and cannot directly attribute the observed improved eGFR slope patterns to the switch from ATV or LPV to DRV.

HIV-positive patients with CKD currently have limited ART options. Tenofovir alafenamide was recently shown to be safe and effective in patients with stable, impaired renal function (creatinine clearance as low as 30 ml/min) [30]. In the absence of randomized clinical trial data on boosted protease inhibitor in renally impaired persons, our data suggest that ATV and LPV may be best avoided in patients with (or at risk of developing) CKD, especially if they require continued use of TDF, and that DRV may be the protease inhibitor of choice for this population.

## Acknowledgements

UK CHIC Steering Committee: Jonathan Ainsworth, Sris Allan, Jane Anderson, Abdel Babiker, David Chadwick, Valerie Delpech, David Dunn, Martin Fisher, Brian Gazzard, Richard Gilson, Mark Gompels, Phillip Hay, Teresa Hill, Margaret Johnson, Sophie Jose, Stephen Kegg, Clifford Leen, Fabiola Martin, Mark Nelson, Chloe Orkin, Adrian Palfreeman, Andrew Phillips, Deenan Pillay, Frank Post, Jillian Pritchard, Caroline Sabin, Achim Schwenk, Anjum Tariq, Roy Trevelion, John Walsh.

UK CHIC Central Co-ordination: University College London (Teresa Hill, Sophie Jose, Andrew Phillips, Caroline Sabin, Susie Huntington); Medical Research Council Clinical Trials Unit at UCL (MRC CTU at UCL), London (David Dunn, Adam Glabay).

UK CHIC Participating Centres: Brighton and Sussex University Hospitals NHS Trust (M Fisher, N Perry, S Tilbury, E Youssef, D Churchill); Chelsea and Westminster Hospital NHS Foundation Trust, London (B Gazzard, M Nelson, R Everett, D Asboe, S Mandalia); King's College Hospital NHS Foundation Trust, London (F Post, H Korat, C Taylor, Z Gleisner, F Ibrahim, L Campbell); Mortimer Market Centre, University College London (R Gilson, N Brima, I Williams); Royal Free NHS Foundation Trust/University College London (M Johnson, M Youle, F Lampe, C Smith, R Tsintas, C Chaloner, S Hutchinson, C Sabin, A Phillips, T Hill, S Jose, A Thornton, S Huntington); Imperial College Healthcare NHS Trust, London (J Walsh, N Mackie, A Winston, J Weber, F Ramzan, M Carder); Barts and The London NHS Trust, London (C Orkin, J Lynch, J Hand, C de Souza); Homerton University Hospital NHS Trust, London (J Anderson, S Munshi); North Middlesex University Hospital NHS Trust, London (J Ainsworth, A Schwenk, S Miller, C Wood); The Lothian University Hospitals NHS Trust, Edinburgh (C Leen, A Wilson, S Morris); North Bristol NHS Trust (M Gompels, S Allan); Leicester, University Hospitals of Leicester NHS Trust (A Palfreeman, K Memon, A Lewszuk); Middlesbrough, South Tees Hospitals NHS Foundation Trust (D Chadwick, E Cope, J Gibson); Woolwich, Lewisham and Greenwich NHS Trust (S Kegg, P Main, Dr Mitchell, Dr Hunter), St George's Healthcare NHS Trust (P Hay, M Dhillon); York Teaching Hospital NHS Foundation Trust (F Martin, S Russell-Sharpe); Coventry, University Hospitals Coventry and Warwickshire NHS Trust (S Allan, A Harte, S Clay); Wolverhampton, The Royal Wolverhampton Hospitals NHS Trust (A Tariq, H Spencer, R Jones); Chertsey, Ashford and St Peter's Hospitals NHS Foundation Trust (J Pritchard, S Cumming, C Atkinson); Public Health England, London (V Delpech); UK Community Advisory Board (R Trevelion).

The UK CHIC Study is funded by the Medical Research Council (MRC) UK (grant numbers G0000199, G0600337, G0900274 and M004236). The views expressed in this manuscript are those of the researchers and not necessarily those of the MRC.

Contributions: Study design: S.J., C.A.S., F.A.P.; data analysis: S.J.; first draft of the manuscript: S.J., F.A.P.; All authors contributed to the data interpretation, final version of the manuscript and approved the submission.

### Conflicts of interest

L.H. was the recipient of a National Institute for Health Research (NIHR) Doctoral Research Fellowship Award. R.J. has received funding to attend conferences or educational meetings, honoraria and/or research grants from Gilead Sciences, Bristol-Myers Squibb, Janssen, GlaxoSmithKline/ViiV Healthcare and Merck. D.C. has received funding to attend conferences or educational meetings, honoraria and/or research grants from Gilead Sciences, GlaxoSmithKline/ViiV Healthcare and Pfizer. C.A.S. has received funding to attend conferences or educational meetings, honoraria and/or research grants from Gilead Sciences, Bristol-Myers Squibb, Janssen, GlaxoSmithKline/ViiV Healthcare and Merck. F.A.P. has received funding to attend conferences or educational meetings, honoraria and/or research grants from Abbvie, Gilead Sciences, Bristol-Myers Squibb, Janssen, GlaxoSmithKline/ViiV Healthcare and Merck. S.J. has received speaker's fees from Gilead Sciences. A.P. declares consultancy and speakers fees from GlaxoSmithKline vaccines and Gilead Sciences and advisory board membership for Abbvie. M.N. has received research grants, travel expenses and honorarium for speaking and advice from GlaxoSmithKline/ViiV Healthcare, MSD, BI, Gilead Sciences, Abbvie and Bristol-Meyers Squib. D.I.W. and R.T. report no conflict of interest.
